# Alzheimer’s Disease: From Pathogenesis to Mesenchymal Stem Cell Therapy – Bridging the Missing Link

**DOI:** 10.3389/fncel.2021.811852

**Published:** 2022-02-07

**Authors:** Jingqiong Hu, Xiaochuan Wang

**Affiliations:** ^1^Stem Cell Center, Department of Cell Therapy, Union Hospital, Tongji Medical College, Huazhong University of Science and Technology, Wuhan, China; ^2^Co-innovation Center of Neuroregeneration, Nantong University, Nantong, China; ^3^Department of Pathophysiology, School of Basic Medicine, Key Laboratory of Education Ministry/Hubei Province of China for Neurological Disorders, Tongji Medical College, Huazhong University of Science and Technology, Wuhan, China

**Keywords:** Alzheimer’s disease, pathogenesis (nervous system), chronic inflammatory response, neurodegeneration, mesenchymal stem cells, stem cell therapeutics, neuroinflammation

## Abstract

Alzheimer’s disease (AD) is the most prevalent neurodegenerative disease worldwide. With the increasing trend of population aging, the estimated number of AD continues to climb, causing enormous medical, social and economic burden to the society. Currently, no drug is available to cure the disease or slow down its progression. There is an urgent need to improve our understanding on the pathogenesis of AD and develop novel therapy to combat it. Despite the two well-known pathological hallmarks (extracellular amyloid plaques and intracellular Neurofibrillary Tangles), the exact mechanisms for selective degeneration and loss of neurons and synapses in AD remain to be elucidated. Cumulative studies have shown neuroinflammation plays a central role in pathogenesis of AD. Neuroinflammation is actively involved both in the onset and the subsequent progression of AD. Microglia are the central player in AD neuroinflammation. In this review, we first introduced the different theories proposed for the pathogenesis of AD, focusing on neuroinflammation, especially on microglia, systemic inflammation, and peripheral and central immune system crosstalk. We explored the possible mechanisms of action of stem cell therapy, which is the only treatment modality so far that has pleiotropic effects and can target multiple mechanisms in AD. Mesenchymal stem cells are currently the most widely used stem cell type in AD clinical trials. We summarized the ongoing major mesenchymal stem cell clinical trials in AD and showed how translational stem cell therapy is bridging the gap between basic science and clinical intervention in this devastating disorder.

## Introduction

Alzheimer’s disease (AD) is the most common neurodegenerative disease. It is characterized by progressive cognitive impairment, disorientation, executive dysfunction, and personality and behavior changes. According to recent World Alzheimer’s Report, there are currently about 50 million people suffering from dementia in the world. With the increasing trend of population aging, it is estimated that by 2050, the total number of people with dementia in the world will reach 152 million (Patterson, 2018). Alzheimer’s disease accounts for more than half of them (Prince et al., 2016).

Alzheimer’s disease is characterized by two pathological hallmarks: amyloid plaques (Aβ) and the neurofibrillary tangles (NFTs). Aβ pathology arises from the improper cleavage of the amyloid precursor protein (APP) resulting in Aβ monomers that aggregate forming oligomeric Aβ and eventually aggregating into Aβ fibrils and plaques (Selkoe, 1994). NFTs are caused by aggregation of hyperphosphorylated Tau protein. Tau is a microtubule-associated protein that stabilizes microtubules (Alonso et al., 1994, 1996, 1997). In AD, tau protein is phosphorylated at multiple sites resulting in dissociation of microtubules from axon and disruption of intracellular trafficking. Furthermore, hyper -phosphorylated Tau forms NFTs which inhibit overall cellular function and eventually lead to neuronal death (Iqbal et al., 1986, 2010).

For decades, the research and development of AD drugs has mainly focused on these two pathological changes, but clinical trials of drugs targeting these two pathologies have all failed, despite being successful in pre-clinical animal models. In general, there is currently no drug that can reverse or alter the course of Alzheimer’s disease. This discrepancy between pre-clinical animal models and clinical translation suggests there is an urgent need to improve our understanding on the pathogenesis of AD.

In this review, we searched the current literature and provided an up-to-date review on the different theories proposed for the pathogenesis of AD, focusing on neuroinflammation, especially on microglia, astrocytes, Natural Killer cells (NK cells), T cells and chronic systemic inflammation. Stem cell therapy holds great promise for AD treatment due to their pleiotropic effects of being able to target multiple pathogenic mechanisms in AD. Mesenchymal stem cells are currently the most widely used stem cell type in AD clinical trials. We summarized the ongoing major mesenchymal stem cell clinical trials in AD and showed how translational stem cell therapy is bridging the gap between basic science and clinical intervention in this devastating disorder.

## Pathogenesis of Alzheimer’s Disease

Different theories have been proposed for AD pathogenesis. In the 1990s, the amyloid cascade hypothesis was the dominant theory, which claims amyloid β is the causative agent in AD and initiates all other AD pathogenesis (Hardy and Higgins, 1992). However, there are several lines of evidence which disagree with this hypothesis: (i) heavy amyloid β loads have been found in many healthy seniors in postmortem brains (Katzman et al., 1988) without causing any cognitive impairment; (ii) amyloid β load is not correlating well with cognitive decline, rather, Tau levels are more relevant (Karran and De Strooper, 2016); (iii) anti-Aβ treatment is ineffective in clinical trials despite clear decrease in patients’ amyloid β load. Later, pathologic Tau theory began to get more popularity. Indeed, hyperphosphorylated Tau proteins are better correlated with cognitive decline. And accumulating data show Tau pathology appears about a decade before amyloid β plaques formation (Arnsten et al., 2021). However, anti-Tau treatment is equally ineffective in clinical trials. In 1987, a seminal study by McGeer et al. (1987) and McGeer and Rogers (1992) aroused researcher’s interest in neuroinflammation. They reported activated microglia in the vicinity of amyloid plaques. To further support their finding, large cohort studies showed the incidence among people using NSAIDs chronically for RA (rheumatoid arthritis) deceased significantly in comparison to normal people (Chang et al., 2016). After that, numerous studies have been focused on inflammation in AD, however, all AD clinical trials using NSAIDs have been proven ineffective so far. Tobin et al. (2019) reported early decrease in neuroblasts of adult neurogenic regions correlates with the extent of cognitive impairment in AD. The impaired neurogenesis occurs prior to amyloid beta accumulation, long before AD clinical manifestation. Since then, there has been a renewed interest in looking at the role of adult hippocampal neurogenesis in the pathogenesis of AD.

**FIGURE 1:**
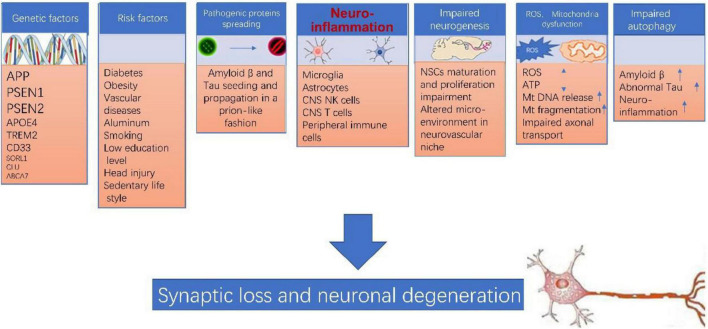
Alzheimer’s disease (AD) pathogenesis. The pathogenesis of AD is extremely complicated, various players such as genetic factor, environment factor, neuroinflammation, pathogenic protein propagation, impaired neurogenesis, mitochondrial dysfunction, ROS accumulation, impaired autophagy likely are all involved in this process, and more importantly, many of these factors interconnect and form a vicious cycle which collectively leads to synaptic loss and neuronal degeneration in AD.

Of note, none of the above theories can explain AD pathogenesis by itself. The pathogenesis of AD is extremely complicated, various players such as genetic factor, environment factor, neuroinflammation, pathogenic protein propagation, impaired neurogenesis, mitochondrial dysfunction, ROS accumulation, impaired autophagy likely are all involved in this process (as illustrated in Figure 1). Many of these factors interconnect and form a vicious cycle which facilitate the damage in neurons. Neuroinflammation likely plays a central role in the pathogenesis of AD (Sarlus and Heneka, 2017; Kinney et al., 2018; Kaur et al., 2019; Onyango et al., 2021).

### Genetic and Environmental Risk Factors

In general, genetic factors do not account for a large proportion of the pathogenesis of neurodegenerative diseases, except for Huntington’s disease (which is entirely inherited). In AD, genetic causes only account for 1–5% of the total disease (Wingo et al., 2012). AD can be divided into early onset and late onset form depending on the age of disease onset. Early onset Alzheimer’s Disease (EOAD) represents less than 10 percent of all patients with Alzheimer’s disease. It typically occurs between a person’s 30s and mid-60s. The three single gene mutations related to EOAD are Amyloid Precursor Protein (APP) on chromosome 21 (Goate et al., 1991), Presenilin 1 (PSEN1) on chromosome 14 (Sherrington et al., 1995), and Presenilin 2 (PSEN2) on chromosome 1 (Rogaev et al., 1995). Most people with AD have the late-onset form of the disease (LOAD), in which symptoms become apparent in their mid-60s and later. Researchers have not found a specific gene that directly causes late-onset Alzheimer’s Disease but a number of risk genes have been identified by large Genome Wide Association Studies (GWAS). APOE ε4 is the most well-known gene that increases the risk of AD. Studies have shown having one or two ε4 alleles of the apolipoprotein E (APOE) gene on chromosome 19 increase a person’s risk to two–fourfold (Blacker et al., 1997). Recent studies have identified other genes which increase the risk of developing Alzheimer’s disease, including TREM2 (Guerreiro et al., 2012), CD33 (Hollingworth et al., 2011), SORL1 (Scherzer et al., 2004), CLU (Harold et al., 2009), ABCA7 (Hollingworth et al., 2011), PINX1 and BIN1 (Harold et al., 2009), etc. TREM2 and CD33 are expressed on microglia of the CNS and play important roles in neuroinflammation, which we will elaborate later. Because genetic form only accounts for a very small percentage of AD, whereas all AD pre-clinical animal models are based on these three genes, one caveat here is that when we used transgenic mouse models to study the disease, the results are not necessarily extrapolated to human studies. In transgenic mice models, treatments targeting Aβ or Tau protein have a significant improvement effect, but these treatments have all failed in human clinical trials. This suggests the limitations of our transgenic animal model (Korhonen et al., 2018; Kolagar et al., 2020). On the other hand, it also shows that patients are extremely heterogeneous in actual clinical practice, and better animal models are needed to address this heterogeneity.

Other risk factors include head injury (Sullivan et al., 1987), aluminum exposure (Hachinski, 1998), vascular disease (Skoog et al., 1999), smoking (Almeida et al., 2008), midlife hypertension (Ou et al., 2020), hypercholesterolemia (Casserly and Topol, 2004), obesity (Grant, 2004), Diabetes Mellitus (DM) (Arvanitakis et al., 2004), sedentary lifestyle (Xu et al., 2013), psychological stress (Sapolsky et al., 1986), low education attainment (Mortimer et al., 2008), etc.

Of all these risk factors, the importance of vascular diseases has gained increased attention. Some AD patients have clear vascular factors, and thus vascular factors have been considered an AD comorbidity. In the seminal study published by Snowdon et al. (1997), it was demonstrated that the presence of lacunar infarcts in the basal ganglia, thalamus or deep white matter causes a reduction in the neuropathological threshold (i.e., senile plaque and NFT load) required for any given grading of AD dementia. After that, accumulating evidences have shown that vascular factors can contribute to AD pathogenesis in various ways. The pathological changes in vessel hemodynamics, angiogenesis, vascular cell function, blood–brain barrier permeability and immune cell migration can affect parenchymal amyloid deposition, neurotoxicity, glial activation and metabolic dysfunction in multiple cell types (Di Marco et al., 2015).

### Impaired Adult Neurogenesis

In adult mammalian brain, there is ongoing adult neurogenesis, which is the process by which neural stem cells produce new neurons. Adult neurogenesis mainly exists in two specific brain areas, namely the subventricular zone (SVZ) of lateral ventricles and the subgranular zone (SGZ) of the dentate gyrus of the hippocampus (Gage, 2000). A third adult neurogenic region in the ventral hypothalamic parenchyma surrounding the third ventricle has also been reported (Kokoeva et al., 2005). Surprisingly, adult neurogenesis persists until the 9th decade of life in normal aging human brains. In AD patients, impaired neurogenesis began very early (Tobin et al., 2019; Moreno-Jimenez et al., 2021). In minimal cognitive impairment (MCI) phase, the number of PCNA + Dcx+ neuroblasts already decreased drastically, and the decrease further deteriorates as AD progresses (Tobin et al., 2019). The decrease in neuroblasts correlates with the extent of cognitive impairment. Of note, the impaired neurogenesis occurs prior to amyloid beta accumulation, long before AD clinical manifestation. Therefore, it is possible that in AD, impairment in neurogenesis, especially in the SGZ neurogenic area, directly affects the learning and memory functions of the hippocampus, and is directly involved in the pathogenesis of AD.

### Autophagy Dysfunction

Accumulating evidence has shown that autophagy is closely related to stem cell aging and neurodegenerative diseases (Li et al., 2017; Kuang et al., 2020). Autophagy is a highly conserved self-repair mechanism in the evolutionary process. Its main function is to degrade longevity proteins in cells and clean up damaged organelles. In AD, Aβ and abnormal phosphorylated Tau protein are usually removed by forming autophagosomes and transferring to lysosomes to form autophagic lysosomes. If autophagy dysfunction leads to unsuccessful removal of these pathological proteins, they will accumulate inside the cells and thus affect neuronal function, and at the same time leading to more autophagy dysfunction (Fleming and Rubinsztein, 2020). This theory has received support from genome wide association studies which identifies CLU gene as a risk factor for LOAD (Harold et al., 2009). CLU is a chaperone protein that participates in autophagosome biogenesis. Furthermore, autophagy is closely linked to neuroinflammation. During early stages of AD, autophagy likely plays positive roles to eradicate Aβ and abnormal phosphorylated Tau protein, however, as AD progresses, autophagy dysfunction will likely exacerbate pathological protein accumulation (François et al., 2014). Targeting autophagy using mTOR might be a new promising strategy to treat AD (Li et al., 2017).

### Pathological Protein Accumulation and Prion-Like Propagation—Seed and Soil Theory

The prion transmission theory of neurodegenerative diseases was initially a controversial theory, but is now widely accepted. This theory states that in each neurodegenerative disease, misfolded protein that adopt an aberrant conformation can provide a template for their own polymerization and thus enable propagation between adjacent cells (Harbi and Harrison, 2014; Walker and Jucker, 2015). As we all know, AD is characterized by presence of hyperphosphorylated Tau and amyloid beta. These pathological proteins, especially amyloid beta, can from prion-like seeds (Prions). These amyloid seeds can continuously replicate, release, and be propagated between adjacent cells in a manner similar to the spread of prion protein lesions. These pathological proteins can also follow the axonal plasm and transported forward or backward to achieve long-distance transmission (Duyckaerts et al., 2019).

### Mitochondrial Dysfunction, Excessive Reactive Oxygen Species Damage

Mitochondria are regarded as the metabolic centers of cells and play pivotal roles in many cell processes, including the immune response. Each mitochondrion contains numerous copies of mitochondrial DNA (mtDNA), a small, circular, and bacterial-like DNA. In response to cellular damage or stress, mtDNA can be released from the mitochondrion and trigger immune and inflammatory responses. Studies have shown excessive ROS and mitochondrial dysfunction are evident in amyloid affected neurons (Tonnies and Trushina, 2017; Singh et al., 2019). Excessive ROS accumulated inside the affected neurons does damage through the following mechanisms: (i) ROS directly do harm to cellular DNA, protein, and membrane (lipid bilayer), destroy their function by causing DNA damage, degradation of cellular protein and lipids; (ii) ROS can induce mitochondrial DNA mutations, damage the mitochondrial respiratory chain, alter membrane permeability, which results in cell energy crisis, mitochondrial transport impairment and mtDNA release; (iii) mtDNA release can further stimulate neuroinflammation (Yoo et al., 2020). ROS damage and mitochondrial dysfunction was once considered the end path in AD, but recent studies have shown excessive ROS is an early event in AD pathogenesis, and play important roles in early neuroinflammation (Tonnies and Trushina, 2017).

## Central Role of Neuroinflammation in Alzheimer’s Disease

**FIGURE 2:**
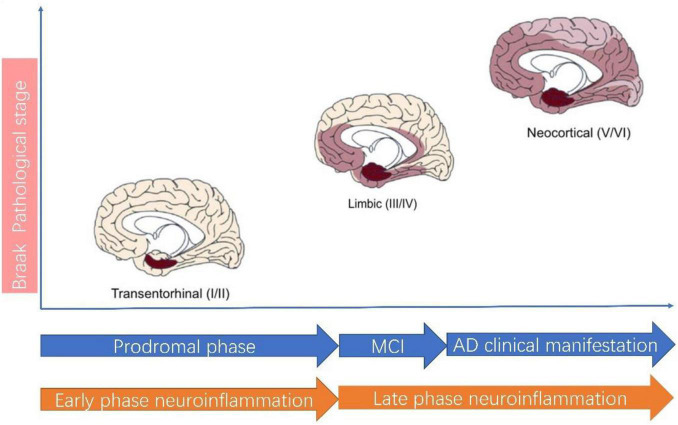
Alzheimer’s disease pathological staging versus clinical staging and neuroinflammation staging. According to AD Braak pathological staging (Braak and Braak, 1991), AD can be staged as I–II: Transentorhinal stages, III–IV: limbic stage and V–VI: Neocortical stage. The Transentorhinal stage roughly corresponds to prodromal phase of clinical AD and Early phase neuroinflammation. In Early phase neuroinflammation, microglia assume a detrimental role. The MCI (mild cognitive impairment) stage of clinical AD roughly corresponds to the beginning of Late phase neuroinflammation, during which the neuroinflammation wanes down.

Neuroinflammation plays a central role in pathogenesis of AD (Heneka et al., 2015; Calsolaro and Edison, 2016; Ahmad et al., 2019; Agrawal and Jha, 2020). According to the classical view, neuroinflammation is characterized by activated microglia and reactive gliosis surrounding the amyloid plaques in AD (Heneka et al., 2015; Calsolaro and Edison, 2016). In this theory, neuroinflammation is considered a passive reaction toward amyloid plaque and Tau protein. Recent studies suggest neuroinflammation actually precedes AD classical hallmarks, which means neuroinflammation is in itself a contributor to AD pathogenesis and represents as the third pathological hallmarks of AD (Cuello, 2017). Thus, the neuroinflammation theory has evolved. A recent review by Cuello (2017) best summarizes this new paradigm shift thinking for neuroinflammation in AD. AD in pathological view needs to be considered as a continuum of disease in which neuroinflammation can be divided into two phases: the early phase and the late phase (Figure 2). The early neuroinflammation phase is the long prodromal phase in AD (can extends for as long as 10–20 years). In this early neuroinflammation phase, a disease-aggravating CNS inflammation predominates and microglia exhibit a pro-inflammatory profile; In late neuroinflammation phase which represents the phase after AD clinical manifestation, the neuroinflammation wanes down to great extent.

The importance of underlining this difference is by employing this new paradigm, we move the timeline of neuroinflammation way ahead to AD prodromal phase. There are several advantages of doing so: first of all, this staging is better in matching Braak pathological stage in AD (Jack et al., 2018; Cummings et al., 2020; Sabbagh, 2020). This also explains why current clinical trials targeting neuroinflammation have all demonstrated lack of efficacy. Most clinical trials recruit only patients with a clear diagnosis of AD, however, even early clinical phase in AD is already in late inflammation pathological phase. This discrepancy in AD clinical phase and pathological phase definition most probably accounts for the failure in using NSAIDs in AD clinical trials. The second advantage is that it improves our awareness for inflammation in AD. It is this early inflammation phase which is more amenable to anti-inflammation therapy but not the latter (Cuello, 2017).

The central player of neuroinflammation is microglia, however, the importance of astrocytes and other immune cells have gained increased attention in recent years.

### Microglia in Alzheimer’s Disease

Microglia are the resident macrophage in central nervous system. Under normal circumstances, resident microglia have a ramified morphology with a small soma, which is described as “resting state” or “quiescent state.” Upon stimulation, microglia are activated and attracted to the site and quickly mount an immune reaction and this is helpful to eradicate amyloid and restrict this inflammation to injury site (Heneka et al., 2015; Kinney et al., 2018; Kwon and Koh, 2020). According to the evolved view of neuroinflammation, the first stimulus for microglia in AD is not amyloid plaques, but rather, amyloid monomers, oligomers or dead neuron debris (Cuello, 2017). After activation, microglia first turned into an anti-inflammatory M2 like phenotype by secreting large amounts of IL-10, TGF-β, IL-4, and IL-13, etc. However, after prolonged activation, they will switch toward a pro-inflammatory M1 like phenotype (Sarlus and Heneka, 2017). The classical M1, M2 classification is actually referring to the peripheral system in which M1 means a pro-inflammatory secreting phenotype for macrophages when they secrete cytokines such as interleukin-1β (IL-1β), tumor necrosis factor-α (TNF-α), and interleukin-6 (IL-6), interleukin-12 (IL-12), etc. M1 is usually associated with decreased phagocytosis activity. M2 means an anti-inflammatory phenotype characterized by secretion of large amounts of anti-inflammatory cytokines such as interleukin-10 (IL-10), tumor growth factor β (TGF-β), and interleukin-4 (IL-4), interleukin-13 (IL-13) etc. M2 is usually associated with increased phagocytosis activity. This arbitrary classification of microglia is only partly true when applied to microglia in the CNS because some microglia have been shown to express both M1 and M2 phenotypes (Heneka et al., 2015) and recent studies have identified new types of microglia which don’t fit in these M1, M2 definitions (Bisht et al., 2016). Here for simplicity reasons, we still use the M1, M2 term, but adding “like” to the term. The role microglia play in AD is more like a double- edge sword. During early neuroinflammation phase, activated microglia assume anti-inflammatory M2 like phenotype by secreting large amounts of IL-10, TGF-β, IL-4, and IL-13, etc. Furthermore, they actively phagocytose amyloid protein, even tau protein. However, prolonged activation of neuroinflammation eventually will stress them out. When they are no longer able to efficiently process large amounts of amyloid load, pathological proteins began to accumulate and aggregate, whereby microglia secret larger amounts of pro-inflammatory cytokines such as IL-1β, tumor necrosis TNF-α, IL-6, and IL-12, etc., similar to M1 phenotype in the periphery (Kwon and Koh, 2020; Onyango et al., 2021). These pro-inflammatory cytokines can direct have impact on APP biosynthesis and lead to further accumulated amyloids and thus form a self-perpetual never-ending vicious cycle, leading to synaptic and neuronal loss in AD. In an APP/PS1 mice AD model, knockout of the NLRP3 inflammasome pathway skews microglia to anti-inflammatory states and protects the mice from memory loss (Heneka et al., 2015).

Microglia also play important role in AD progression. Studies have shown microglia are primed toward a chronic inflammatory response activation state. This chronic inflammatory response activation state is specifically manifested by the reduction of the excitability threshold of microglia and the long-term sustained secretion of low-level pro-inflammatory factors such as TNFα, IL-6, IL-1, etc. (Sarlus and Heneka, 2017).

Recent studies have shown microglia in the CNS are heterogenous and may exhibit a variety of phenotypes with distinct functions depending on location, activation state and host environment. For example, a new phenotype of microglia, referred to as “dark microglia,” was found in conditions such as chronic stress, including in the APP/PS1 mouse model of AD. Notably, dark microglia exhibited a highly activated phenotype with strong expression of CD11b and TREM2 and extensive encircling of synaptic clefts when the microglia were associated with amyloid deposits (Bisht et al., 2016). Disease-associated microglia (DAM) are a subset of microglia showing a unique transcriptional and functional signature (Keren-Shaul et al., 2017; Deczkowska et al., 2018) recently identified by comprehensive single-cell RNA analysis of CNS immune cells in neurodegenerative conditions. These microglia display a dedicated sensory mechanism to detect neural tissue damage in the form of neurodegeneration-associated molecular patterns (NAMPs), a model analogical to the peripheral immune system’s pathogen- and damage-associated stress signals (PAMPs and DAMPs) (Chiarini et al., 2020).

### Astrocytes

Astrocytes are the most abundant cell type in the central nervous system. They provide structure, metabolic and neurotrophic support for normal function of neurons. The roles astrocytes played in neuro-inflammation have been increasingly recognized. Astrocyte accumulation in the vicinity of amyloid plaques is among the earliest pathological events in AD patients and animal models (Heneka et al., 2015). These astrocytes are referred as “reactive astrocytes” and they undergo morphological and transcriptional changes including ramification of hypertrophic processes and increased GFAP (glial fibrillary acidic protein) expression. Astrocytes express multiple genes related to AD, including Apolipoprotein E (APOE), Clusterin (CLU) and Fermitin family member 2 (FERMT2) (Preman et al., 2021). Astrocytes also express enzymes which contribute to the degradation of β-amyloid. These enzymes include neprilysin (NEP), insulin-degrading enzyme (IDE), and endothelin-converting enzymes 1 and 2 (ECE1 and ECE2) (Preman et al., 2021). Furthermore, astrocytes express aquaporin 4 (AQP4) water channels in their vascular end-feet and play an essential role in the glymphatic system implicated in the clearance of beta-amyloid (Ries and Sastre, 2016). Upon stimulation, they first assume an anti-inflammatory role by secreting of TGF-β, enhancing phagocytosis of dystrophic neurites and synapses. This phenotype is referred as the A2 phenotype, similar to the anti-inflammatory M2 phenotype of microglia (Ben Haim et al., 2015). However, prolonged activation eventually renders them to exert detrimental effects by producing pro-inflammatory cytokines such as TNF-α, IFN-γ, IL-1β, and cyclooxygenase-2 (COX-2), increased ROS, as observed in several AD mouse model studies (Ben Haim et al., 2015; Heneka et al., 2015). This phenotype is referred as the A1 phenotype. It is generally believed in neuroinflammation, astrocytes and microglia both work in concert. However, microglia likely provide signals to induce astrocytes into reactive phenotype (Liddelow et al., 2017). Despite the common impression of hypertrophy for reactive astrocytes, studies have shown in many parts of the brain in AD, there are atrophic astrocytes (Ben Haim et al., 2015). Atrophic astrocytes are characterized by reduced volume and thinner processes as revealed by morphometric analysis of cells immunolabelled with antibodies against GFAP and S100β. In the 3xTg-AD mice model, atrophic astrocytes appear as early as 1-month age in the entorhinal cortex (EC) and the atrophy is sustained after 12 months of age when β-amyloid plaques begin to appear. Similar to microglia, recent transcriptome study and single cell RNA sequencing technologies also identify heterogeneity within astrocytes population. Furthermore, they likely play dynamic roles in different stages of AD. Recently, Chun et al. (2020) developed an intricate model system which can modulate the degree of astrocytes activation *in vivo*. Using this system, they showed dysfunctional astrocytes can be divided into mild-moderate reactive astrocytes and severe reactive astrocytes. Mild reactive astrocytes can naturally reverse their reactivity, whereas severe reactive astrocytes have no chance to reverse their reactivity and they can lead directly to tauopathy, neuronal death, brain atrophy, cognitive impairment and eventual death in APP/PS1 AD mice model (Chun et al., 2020). Their finding suggests severe reactive astrocytes can cause neurodegeneration alone, independent of microglia, which adds more complexity to their elusive role in neuroinflammation.

### Natural Killer Cells and Activated T Cells

Normally NK cells and activated T cells are not seen in central nervous system due to brain–blood barrier (BBB). In the case of acute or chronic brain injury, studies have shown in neurodegenerative diseases such as AD, BBB permeability is increased (Ennerfelt and Lukens, 2020). Peripheral immune cells can thus enter the brain through the leaky blood brain barrier, which we will elaborate later. With the advent of single cell sequencing technology, we are beginning to discover more than we previously anticipated. Recent studies have shown, within central nervous system, there are resident NK cells and T cells in AD patients. Jin et al. (2021) reported recently that NK cells of the innate immune system reside in the dentate gyrus neurogenic niche of aged brains in humans and mice. Neuroblasts within the aged dentate gyrus display a senescence-associated secretory phenotype and reinforcement of NK cell functions results in NK cell mediated elimination of aged neuroblasts. These results demonstrate that resident NK cells accumulation in the aging brain impairs neurogenesis, which may serve as a therapeutic target to improve cognition in the aged population. In another study, Zhang Y. et al. (2020) reported that depletion of NK cells alleviates neuroinflammation, stimulates neurogenesis, and improves cognitive function in a triple-transgenic AD mouse model. NK cells in the brains of triple-transgenic AD mouse model (3xTg-AD) mice exhibited an enhanced proinflammatory profile. Depletion of NK cells by anti-NK1.1 Abs drastically improved cognitive function of 3xTg-AD mice. In 3xTg-AD mice depleted of NK cells, microglia demonstrated a homeostatic-like morphology, decreased proliferative response and reduced expression of proinflammatory cytokines. Together, these results suggest a proinflammatory role for NK cells in AD and depleting of NK cells can alleviate inflammation and increase cognitive function in AD mice or patients. Notably, NK cells act not through amyloid β but through enhanced neurogenesis and alleviated inflammation.

In another single cell sequencing study, analysis of 14,685 single cell transcriptomes in aged mice brain reveals a decrease in activated NSCs, changes in endothelial cells and microglia, and infiltration of CD8+ T cells in SVZ neurogenic niches (Dulken et al., 2019). Notably, these T cells are clonally expanded which suggest that they have come across some specific antigen in the brain, and not just infiltrate from compromised BBB. These T cells express interferon γ, and the subset of NSCs with a high interferon response shows decreased proliferation *in vivo*. Single cell sequencing technology opens new avenue for our understanding of immune system in the brain. The brain used to be considered an immune-privileged organ, however, this concept has changed and now we know in neurodegenerative diseases such as AD, not only does brain’s innate immune system play a role, but also its adaptive immune system.

### Peripheral Immune Reaction

**FIGURE 3:**
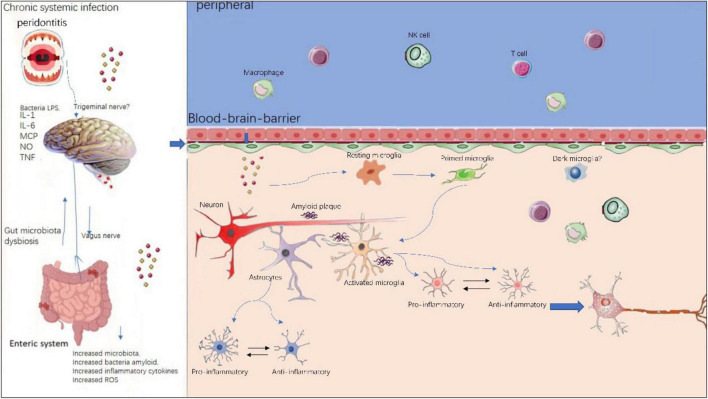
Peripheral and central immune system crosstalk in AD. Chronic systemic infections such as periodontitis and gut microbiota dysbiosis will produce local infection and increased inflammatory cytokines and peripheral immune cells such as peripheral microphages and T cells, NK cells are able to enter the compromised blood–brain-barrier and exacerbate existing neuroinflammation in central nervous system. Within the brain, resident microglia transform from resting state to activated state upon stimulation. Initially, microglia and astrocytes exhibit anti-inflammatory phenotypes, however, sustained chronic inflammation will drive them toward pro- inflammatory phenotypes.

The neuroinflammation in AD has long been considered a local immune reaction restricted to the CNS, the role that peripheral immune reaction played in this process has been less investigated. However, a growing body of evidence suggests peripheral immune cells play a vital part in the pathogenesis of AD, as illustrated in Figure 3. In neurodegenerative diseases, BBB is compromised and thus enabling crosstalk between peripheral and central nervous system (Rosenberg, 2012). In AD patients and transgenic animal models, altered levels of pro-inflammatory cytokines in blood have been frequently reported (Bettcher et al., 2018; Meyer et al., 2018). Recently, Xu and Jia (2021) reported a single-cell transcriptome study from AD patients. They profiled 36,849 peripheral blood mononuclear cells from AD patients with amyloid-positive status and normal controls with amyloid-negative status. They could identify five immune cell subsets: CD4+ T cells, CD8+ T cells, B cells, natural killer cells, and monocytes-macrophages cells. They also found high-frequency amplification clonotypes in T and B cells and decreased diversity in T cells in AD. These findings suggest that the peripheral adaptive immune response, especially mediated by T cells, may have a role in the pathogenesis of AD.

Peripheral immune cells contribute to AD pathogenesis via several mechanisms: (i) peripheral immune cells such as T cells and B cells, NK cells can enter the brain via BBB, thereby aggregating existing local immune reaction mediated by microglia; (ii) the pro-inflammatory cytokines secreted by peripheral immune cells can also penetrate the brain via BBB, thus integrating into the cytokine network in CNS. On the other hand, there is evidence of the effect of regional neuroinflammation on peripheral immune processes. This means the peripheral and the central immune system is not a one-way street, but rather constantly exchanging (Bettcher et al., 2021).

This new concept allows us to consider the characteristics of immune status as informative indicators for early detection of the AD development and for the modulating of immunotherapeutic approaches in AD.

### Systemic Inflammation in Alzheimer’s Disease

Cumulative evidence has shown systemic inflammation plays a role in neurodegenerative diseases (Bettcher et al., 2021). Of these systemic inflammation situation, two kinds of inflammation have gained particular interest in recent years. One is periodontitis and the other is gut microbiota dysbiosis (Teixeira et al., 2017; Megur et al., 2020). Recently, attention has been focused on the relationship between periodontitis and AD. *Porphyromonas gingivalis* (*P. gingivalis*) and its toxins have been detected in autopsy brain tissues from patients with AD (Teixeira et al., 2017) but absent in normal patients. *P. gingivalis* is from a Gram-negative periodontal pathogen, *Porphyromonas gingivalis* (Pg) and/or its product gingipain is/are translocated to the brain. Ilievski et al. (2018) proved repeated exposure (22 weeks) of wild type C57BL/6 mice to orally administered Pg results in neuroinflammation, neurodegeneration, microgliosis, astrogliosis and formation of intra- and extracellular amyloid plaque and neurofibrillary tangles (NFTs) which are pathognomonic signs of AD (Ilievski et al., 2018). In addition, Pg/gingipain was detected in the hippocampus of mice in the experimental group by immunohistochemistry, confocal microscopy, and qPCR confirming the translocation of orally applied Pg to the brain. Thus this study is the perfect example for using Koch’s postulates to prove a microorganism’s contribution to a disease. The only caveat is that according to Koch’s postulates, the microorganism has to be cultured directly in affected animals. This study is the first to show neurodegeneration and the formation of extracellular Aβ42 in young adult WT mice after repeated oral application of Pg. The neuropathological features observed in this study strongly suggest that low grade chronic periodontal pathogen infection can result in the development of neuropathology that is consistent with that of AD.

In periodontal disease, systemic inflammation affect AD via two mechanisms: (i) the periodontal bacteria (especially Gram-negative, LPS containing bacteria in the biofilm of periodontal pockets) can produce proinflammatory cytokines locally and these cytokines enter CNS through defect BBB (Teixeira et al., 2017); (ii) In AD, the microglia are already primed toward a pro-inflammatory phenotype (Heneka et al., 2015). Any further inflammatory trigger can intensify the microglia response and exacerbate existing inflammation. Thus periodontitis is believed to play a role in AD progression. There are also studies reported periodontitis may even play important role in AD initiation (Dioguardi et al., 2020).

The intestinal microflora takes part in bi-directional communication between the gut and the brain. Gut microflora may even act as the “second brain.” They are able to produce several neurotransmitters and neuromodulators like serotonin, kynurenine, catecholamine, etc. (Carranza-Naval et al., 2021). Bacteria populating the gut microbiota can also produce large amounts of amyloids and lipopolysaccharides. Alterations in the gut microbiota composition can (i) induce increased permeability of the gut epithelial barrier (Megur et al., 2020); (ii) bacterial amyloids can break the gut barrier into blood stream, finally enter the brain through the leaky blood–brain-barrier (or via olfactory bulb), where they prime residential microglia through molecular mimicry (Friedland, 2015) and promote neuroinflammation, neural injury, and ultimately neurodegeneration. This theory is relatively new and so far only evidence in animal studies. However, it did raise some hope for treating AD using modulation of gut microbiota such as using probiotics or FMT (Fecal Microbiota Transplantation).

Other studies have shown a connection for HSV infection and AD (Laval and Enquist, 2021). At the same time, a chronic inflammatory state has been shown for AD and was believed to be directly involved in the progression of AD (Kwon and Koh, 2020).

## Mechanisms of Neuronal and Axonal Degeneration via Neuroinflammation—A Multi-Step Process

How does amyloid accumulation and neuroinflammation lead to neuronal degeneration and axonal degeneration? The exact mechanism remains to be elucidated. It has been shown in the beginning of AD, amyloid plaques accumulated in various sites of the brain are actually not influencing directly in cognitive function. However, as AD progresses, when synapses are affected and axonal transport is hindered at multiple sites along the axon due to false phosphorylated Tau protein, the neurons will degenerate. Recently, FA Edwards (2019) brought up an elegant hypothesis for amyloid induced neurodegeneration. His hypothesis brings together a wide range of evidence from different laboratories, to address how deposition of amyloid β, once initiated, eventually leads to Tau tangles and neurodegeneration. He defines the neuronal degeneration in AD into four steps: (i) accumulation of microglia and astrocytes to amyloid plague; (ii) restriction of neuroinflammation; (iii) synapses are affected along multiple sites of axon; (iv) degeneration of neurons and axons irreversible.

In this hypothesis, he emphasized the important role of microglia. He proposed the efficiency of microglia to eliminate amyloid load determines the progression speed in AD. In healthy seniors, amyloid β is frequently detected postmortem. Although amyloid β is the trigger for neuroinflammation, studies have shown it is not corresponding to synaptic loss and neuronal neurodegeneration. His proposed theory explains that only when a neuron encounters multiple along its trajectory route, did this become a serious problem. Third, it explains the difference in individuals to AD lies in one’s efficiency of microglia to eliminate amyloid load determines the progression speed in AD.

However, this theory doesn’t explain the signaling pathways how degeneration of neurons and axons occur. Amyloid signaling pathways include the glycogen synthase kinase-3β, nuclear factor kappa B cascade, mitogen-activated protein kinase pathways and c-Jun N-terminal kinase (Chiarini et al., 2020). Amyloid initiate innate immune system through binding to TREM and TLR receptors on microglia. TREM2 is a type-1 transmembrane glycoprotein predominantly expressed in microglia. TREM2-ApoE interaction is essential for the phagocytosis of apoptotic neurons and extracellular debris, from dying or dead neurons. TLRs are a superfamily of pattern recognition receptors and remain the front-line defense against pathogenic infection and tissue injury. TLR-2 activation triggers Aβ-induced inflammation via NF-κB pathway (Gambuzza et al., 2014). TLR-4 have been reported to initiate the inflammatory pathway cascade via mitogen-activated protein kinase (MAPK) and c-Jun N-terminal kinase pathway (Walter et al., 2007; Calvo-Rodriguez et al., 2020). Stimulation of TLRs on microglia can further mount an inflammatory response by the NRLP3 (NACHT, LRR, and PYD domains-containing protein). In an APP/PS1 mice AD model, knockout of the NLRP3 inflammasome pathway skews microglia to anti-inflammatory states and protects the mice from memory loss (Heneka et al., 2015). As a consequence of the NRLP3 inflammasome activation, caspase-1 is recruited to the inflammasome, and a number of proinflammatory mediators such as TNFα and IL-1β are released to induce further pro-inflammatory responses. Hence this neuroinflammatory reaction forms a vicious cycle, triggering further neuronal loss in AD.

Recently, necroptosis, a programed form of necrosis has been proven in postmortem human AD brains (Caccamo et al., 2017; Jayaraman et al., 2021). Unlike apoptosis, necroptosis is executed by the mixed lineage kinase domain-like (MLKL) protein, which is triggered by receptor-interactive protein kinases (RIPK) 1 and 3. Necroptosis was found to be activated in postmortem human AD brains, positively correlated with Braak stage, and inversely correlated with brain weight and cognitive scores (Caccamo et al., 2017). This necroptosis is likely linked to TNF (tumor necrosis factor) -mediated signaling pathway because increased expression of multiple proteins linked to TNF signaling pathway can be predominantly observed in the CA1 pyramidal neurons in the AD post-mortem brain (Jayaraman et al., 2021), accompanied by phosphorylation of RIPK3 and MLKL. These findings suggest targeting TNF-mediated necroptosis might be potential targets in AD pathogenesis.

## Factors Contribute to the Sustained Progression of Alzheimer’s Disease

Alzheimer’s disease has an extremely long latent period (10–20 years) before clinical manifestation (Calne et al., 1986). For AD to be slowly progressive, three factors play important roles: aging status of the host, chronic systemic inflammation and microglia dysfunction.

### Aging Status of the Host

Various studies have shown aging is not only an extremely important risk factor for AD, but also play important roles in the progression of AD. Studies have shown intracerebral injection of beta amyloid is less likely to induce AD in young mice in comparison to old mice (Kane et al., 2000). In aged brain, aging cells feature a decreased ability to proliferate, whereas keep the basic metabolism level and this phenomenon is termed “senescence” (Muñoz-Espín and Serrano, 2014; Kirkland and Tchkonia, 2017). This aging affects neurons, microglia, astrocytes and also neural stem cells in adult neurogenic zones. But most importantly, aging affects our immune system as a whole. Inflammaging and immunosenescence are the two hallmarks of biological aging (Thomas et al., 2020). The term “Inflammaging” was first coined by Franceschi et al. (2007). It refers to the age-related increase in pro-inflammatory mediators in peripheral blood, which is due to senescence induced secretion of pro-inflammatory cytokines, causing a state of chronic inflammation without overt infection (“Sterile” inflammation). This means in aged brain, microglia are already primed toward pro-inflammatory phenotype (Giunta et al., 2008; Heneka et al., 2015; Sarlus and Heneka, 2017).

Aging not only affects the immune system, it also leads to increased oxidative stress (Benz and Yau, 2008). The imbalance between the production of ROS and antioxidants, deteriorates considerably with aging. ROS can directly harm microglia, astrocytes, and neurons (Zuo et al., 2019). ROS can also affect mitochondria function in microglia, astrocytes and neurons. This leads to release of mitochondria cyclic DNA directly to blood stream, exacerbating existing neuroinflammation (Hoye et al., 2008).

In aged brain, there is a decreased endogenous neural stem cell pool. NSCs exhibiting a cellular senescence phenotype have been observed in the dentate gyrus of the APP/PS1 transgenic mouse AD model (Daynac et al., 2014). The neurovascular niche of neural stem cells also shows senescence features in aged brain (Navarro Negredo et al., 2020). Jin et al. (2021) reported elimination of senescent neuroblasts improve cognitive function in transgenic AD mice model. Inability to replenish adult progenitor cells due to cellular senescence could render the central nervous system susceptible to neurodegeneration.

The blood–brain-barrier is also compromised is aged brain, enabling entry of peripheral immune cells. Vascular cells and specifically endothelial cells and pericytes have been shown to undergo senescence *in vitro* and *in vivo* (Tarantini et al., 2017; Huang Z. et al., 2020). Accumulation of senescent endothelial cells is associated with impaired tight junction structure and compromised blood–brain barrier integrity (Yamazaki et al., 2016).

Last but not the least, autophagy is diminished in aged brain (Berglund et al., 2020), further affecting pathological protein eradication.

### Chronic Systemic Inflammation

It’s very common for AD patients to have co-existing chronic systemic inflammation. Importantly, chronic systemic inflammation usually synergizes with inflammaging. For example, in periodontal disease, the periodontal bacteria (especially Gram-negative, LPS containing bacteria in the biofilm of periodontal pockets) can produce proinflammatory cytokines locally and these cytokines enter CNS through defect BBB, where they meet the microglia, which are already primed toward a pro-inflammatory phenotype. This additional inflammatory trigger can intensify the microglia response and exacerbate existing inflammation. Fielder et al., reported using a mouse model of genetically enhanced NF-κB activity (nfκb1–/–), characterized by low-grade chronic inflammation and premature aging, to investigate the impact of chronic inflammation on cognitive decline. They found that during aging, nfkb1–/– mice show an early onset of memory loss, combined with enhanced neuroinflammation and increased frequency of senescent cells in the hippocampus and cerebellum (Fielder et al., 2020). Importantly, treatment with the non-steroidal anti-inflammatory drug (NASID) ibuprofen reduced neuroinflammation and senescent cell burden resulting in significant improvements in cognitive function. These data provide evidence that chronic inflammation is a causal factor in the cognitive decline observed during aging.

Chronic inflammatory state can directly influence cognitive function. Studies have shown AD patients tend to deteriorate more quickly under a chronic inflammatory state (Teixeira et al., 2017).

### Microglia Dysfunction

It has been shown by post-mortem brain autopsy that in many healthy seniors there are as many amyloid plaques as in AD patients, without causing any cognitive dysfunction (Katzman et al., 1988). To explain this discrepancy, Edwards (2019) has put up an elegant hypothesis. He hypothesized this different outcome is due to the difference in the ability of microglia to eliminate amyloids. In people with normal microglia function who can efficiently remove amyloid plaques, the onset and progression of AD will be postponed. On the contrary, in people with abnormal microglia function who can’t efficiently remove amyloid plaques, a relatively lower plaque load and a relative shorter latency period is sufficient for elicit cognitive decline and eventually AD. But what are the factors contributing the major difference in efficiency of microglia to clear amyloid plaques? Studies have shown this is due to the different expression of TREM2 and CD33 genes on their surface. It has been suggested that the TREM2 is the major positive regulator of microglial phagocytosis whereas CD33 is the key negative regulator of this process (Griciuc et al., 2019). Triggering receptor expressed on myeloid cell 2 (TREM2) is a transmembrane receptor of the immunoglobulin superfamily expressed on the plasma membrane of myeloid cells and microglia, and is active in the innate immune response. TREM2 is a major risk gene identified by genome wide association studies in AD (Gratuze et al., 2018). It can bind to Lipopolysaccharides (LPS), phospholipids, HDL, LDL, APOE, apoptotic neurons, and Aβ (Wolfe et al., 2018), all of which activate signaling pathways. It seems TREM2 is required for microglia to convert from a homeostatic profile to a DAM profile (Ulland and Colonna, 2018). Wang S. Y. et al. (2020), Wang Y. et al. (2020) found that knockout of Trem2 in a 5xFAD mouse model led to exacerbation of AD, with an increased burden of Aβ plaques in the hippocampus due to a dysfunctional response of microglia, in which they fail to accumulate around Aβ plaques.

For AD to be slowly progressive, the above three factors interact with each other and form a vicious cycle which amplifies the existing neuroinflammation and synaptic and neuronal loss. Aging status is usually accompanied by persisting systemic inflammation; chronic inflammation exacerbates cellular senescence and overall immune status of the patient; dysfunctional microglia further deteriorate chronic inflammation and senescence.

## From Pathogenesis to Therapy: Mesenchymal Stem Cell Therapy for Alzheimer’s Disease

Currently, two types of drugs are commonly used to treat Alzheimer’s disease. One is anticholinesterase drugs, including galantamine and Donepezil; the other is excitatory glutamine acid NMDA receptor antagonists, such as Memantine. These drugs are only used for the symptomatic treatment. So far there is no disease-modifying therapies (DMTs) that can halt or reverse the disease process. DMTs clinical trials are arduous. The majority of DMTs clinical trials are aimed at the two classic pathological hallmarks, namely, amyloid and Tau, however, these trials have all failed (Cummings et al., 2020). Recently, the United States FDA adopted an accelerated approval for Biogen’s Aduhelm (aducanumab, an amyloid antibody) for the treatment of AD. However, this approach caused a lot of controversy, because in phase 3 clinical trial, its effect is controversy.

The reason for failure in AD therapy is because the exact mechanisms for selective neuronal loss remains to be elucidated. Current drug clinical trials employ “one drug, one mechanism” concept. AD pathogenesis is extremely complicated and only targeting those single pathological feature such as amyloid beta, Tau, or neuroinflammation is not likely to achieve clinical success. Furthermore, current preclinical animal models are all based on transgenic mice, whereas hereditary form of AD only represents less than 5% of the disease. Therefore, direct translation of results in animal models to clinical therapy is not always feasible. Stem cells are a type of cells with self-renewal and multi-lineage differentiation potential. Stem cells can promote the regeneration and repair of the nervous system, and the core pathological feature of neurodegenerative diseases is the selective loss of neurons, which makes stem cell therapy the ideal treatment option. Stem cell therapy is the only treatment modality which can target multiple mechanisms in AD and can possibly lead to positive results without knowing the exact mechanisms behind the disease. Ever since the first stem cell therapy attempt using human fetal midbrain tissue to treat Parkinson’s disease patient in 1980, four decades have passed. At present, many types of stem cells have been studied in the treatment of AD, including embryonic stem cells (ESCs), neural stem cells (NSCs), mesenchymal stem cells (MSCs), and induced pluripotent stem cells (iPSCs), etc. (Sivandzade and Cucullo, 2021). Mesenchymal stem cells (MSCs) are the most widely used stem cells in AD clinical trials.

Mesenchymal Stem Cells or Mesenchymal Stromal Cells were first discovered by Friedenstein et al. (1974) as adhering cells growing in whirlpool like morphology in the bone marrow. Since then, MSCs have been isolated from various sources such as the adipose tissue, umbilical cord and cord blood, placental tissue, amnion fluid, even the dental pulp (Duncan and Valenzuela, 2017). MSCs can be effectively induced into bone, cartilage, fat, and muscle and even neurons *in vitro*. Dominici et al. (2006) (i) adhere to plastic surface; (ii) express high levels of CD105, CD73, CD90, and lack expression (<2% positive) of CD45, CD34, CD14 or CD11b, CD79α or CD19 and HLA class II; (iii) *in vitro* differentiation into osteocytes, adipocytes and chondrocytes. Nonetheless, MSCs are heterogenous in nature. Studies have shown different subset of MSCs actually have different self-renewal capacity and also varies in their osteogenic, adipogenic, chondrogenic and neurogenic capacity. These differences are likely attributed to their tissue origins.

Mesenchymal stem cells have many advantages: (i) they aren’t involved in any complicated ethical issues as with ESCs and NSCs; (ii) they are easy to obtain, easy to manipulate, easy to stock; (iii) they almost express no HLA antigen and therefore allogeneic transplantation can be achieved without immunosuppression; (iv) they are less prone to tumor formation; (v) MSCs can modulate immune reaction and alleviate neuroinflammation in AD. These amenable features make MSCs currently the most widely used stem cell source in regenerative therapy for AD.

### Proposed Mechanisms of Action of Mesenchymal Stem Cells Therapy in Alzheimer’s Disease

Different mechanisms have been proposed for MSCs therapy in AD. The main mechanisms of action of MSCs therapy have long been considered to be “cell replacement.” But increasing evidence have shown transplanted MSCs can only survive in the host for a very limited amount of time. Intravenously administered MSCs mostly are trapped in the lung and the spleen (Park B. N. et al., 2018; Kim et al., 2020; Park et al., 2020; Hernandez and Garcia, 2021).

Now it is generally believed transplanted MSCs mainly function through paracrine effects (Walker and Jucker, 2015; Duncan and Valenzuela, 2017; Guo et al., 2020; Kim et al., 2020; Liu et al., 2021). We and others have shown mesenchymal stem cells secrete a large number of neurotrophic and angiogenic factors through paracrine action, especially glial cell derived neurotrophic factor (GDNF), vascular endothelial growth factor (VEGF), brain-derived neurotrophic factor (BDNF), and insulin growth factor (IGF), etc. These neurotrophic and angiogenic factors can potentially improve the microenvironment for the surviving neurons in the diseased area, and promote neuron regeneration and repair. Santamaria et al. (2021) recently reported intranasal administration of secretome collected from MSCs exposed *in vitro* to AD mouse brain homogenates (MSCCS) induced persistent memory recovery, with dramatic reduction in amyloid plaque load and reactive gliosis in APP/PS1 AD mice model. They also found a higher neuronal density in cortex and hippocampus, associated with a reduction in hippocampal shrinkage and a longer lifespan indicating healthier conditions of MSC-CS-treated compared to vehicle treated APP/PS1 mice. This suggests the positive effects associated with MSCs transplantation in AD such as improvement on memory, accelerated amyloid plaque clearance, alleviated neuroinflammation and stimulation of endogenous neurogenesis can all be mimicked using MSCs derived secretome, which strongly indicates that the paracrine effects of MSCs play an important role in MSCs transplantation studies.

Another important mechanism for MSCs therapy is modulation of neuroinflammation. As aforementioned, neuroinflammation plays pivotal roles in the pathogenesis of AD. Multiple studies have shown mesenchymal stem cells can convert microglia and astrocytes from pro-inflammatory phenotypes M1 and A1 to anti-inflammatory phenotypes M2 and A2, thereby alleviating the neuroinflammatory response and neuronal damage in AD (Wei et al., 2018; Zhao et al., 2018; Qin et al., 2021). Zhao et al. (2018) recently showed that intracerebral transplantation of menstrual blood- derived MSCs dramatically improved the spatial learning and memory of APP/PS1 mice. The expression of proinflammatory cytokines were remarkably reduced and they suggest the switching of microglia from pro-inflammatory phenotype to anti- inflammatory phenotype likely explains the positive effects. Xie et al. (2016) reported intravenously transplanted Wharton’s Jelly MSCs significantly improved the spatial learning and alleviated the memory decline in the APP/PS1 mice. Aβ deposition and soluble Aβ levels were significantly reduced after WJ-MSCs treatment. WJ-MSCs significantly decreased the expressions of pro-inflammatory cytokines IL-1β and TNFα and at the same time, increased the expression of the anti-inflammatory cytokine IL-10. In another study, Ma et al. (2013) transplanted Adipose derived MSCs (Ad-MSCs) intracerebrally into APP/PS1 transgenic mice. Ad-MSCs dramatically reduced β-amyloid (Aβ) peptide deposition and significantly restored the learning/memory function in these mice. It was observed that in both regions of the hippocampus and the cortex there were more activated microglia, which preferentially surrounded and infiltrated into plaques after ADSC transplantation. The activated microglia exhibited an activated phenotype, as indicated by their decreased expression levels of proinflammatory factors and elevated expression levels of anti-inflammatory factors, as well as Aβ-degrading enzymes. These studies suggest MSCs transplantation can alleviate cognitive decline in AD mice through anti-neuroinflammation mechanisms.

Recent studies have shown early in AD, there is impaired endogenous neurogenesis and decrease neural stem cell pool in adult hippocampus neurogenic regions (Tobin et al., 2019; Moreno-Jimenez et al., 2021). MSCs transplantation can potentially take effect by replenishing the endogenous neural stem cell pool and stimulating neurogenesis. Yan et al. (2014) reported increased neurogenesis in SVZ and SGZ hippocampal regions after adipose tissue derived MSCs transplantation in APP/PS1 mice model.

The MSCs can also directly target classical AD hallmarks. Multiple studies using MSCs transplantation in AD transgenic mice models have shown reduced Aβ plaque burden and decreased levels of tau hyperphosphorylation (Naaldijk et al., 2017; Zhao et al., 2018; Santamaria et al., 2021). Soluble intracellular adhesion molecule-1 (sICAM-1) secreted by umbilical cord derived MSCs can induce the expression of neprilysin (a Aβ-degrading enzyme) and thus facilitate Aβ clearance. MSCs can further reduce Aβ plaque burden through internalization and Aβ degradation of endosomal–lysosomal pathway. In animal studies, MSCs transplantation have been shown to improve the symptoms of AD rats by accelerating the clearance of amyloid and tau (Kim et al., 2012). Zhao et al. (2018) reported Menstrual blood derived MSCs transplantation dramatically reduced tau phosphorylation at Ser202/Thr205 (AT8) and Ser396 sites in the brains of APP/PS1 mice.

Mitochondrial transfer as a new mechanism of stem cell therapy has attracted wide attention and has been considered as a potential therapy for tissue damage. Studies have shown that mesenchymal stem cells may transfer their healthy mitochondria to dying neurons to restore energy metabolism and save neurons (Hayakawa et al., 2016). Mitochondrial transfer can be achieved via extracellular vesicle (EV), TNT (tunneling nanotube) or cell fusion (Wang and Gerdes, 2015; Liu et al., 2019; Gomzikova et al., 2021). Although so far mainly proven in brain ischemia animal models (Huang L. et al., 2020), it might also play a role in AD. Recently, Zhang L. et al. (2020) reported human umbilical cord mesenchymal stem cells (hucMSCs) can donate healthy mitochondria to okadaic acid (OA)-treated SH-SY5Y cells and restore their mitochondria function in an AD cell model (Zhang Z. et al., 2020).

Other mechanisms being implicated for MSCs therapy in AD include enhancing autophagy, decrease ROS (Guo et al., 2021), renormalization of BBB and NVU (neural-vascular unit).

Exosomes have drawn much attention in the stem cell therapy field recently. MSCs derived exosomes are enriched in neurogenic and angiogenic cytokines, mRNA and microRNA. These substances can be secreted and transferred to other cells or used to control the surrounding microenvironment. Exosomes can be transferred to target cells through the BBB without being degraded in blood because they are surrounded by lipid bilayer (Chen et al., 2021). Recently, an exosome- based mesenchymal stem cell trial is launched in China. Nasal inhalation of exosomes of allogeneic adipose-derived mesenchymal stem cells is used for the treatment of mild to moderate AD patients (NCT04388982).

**FIGURE 4:**
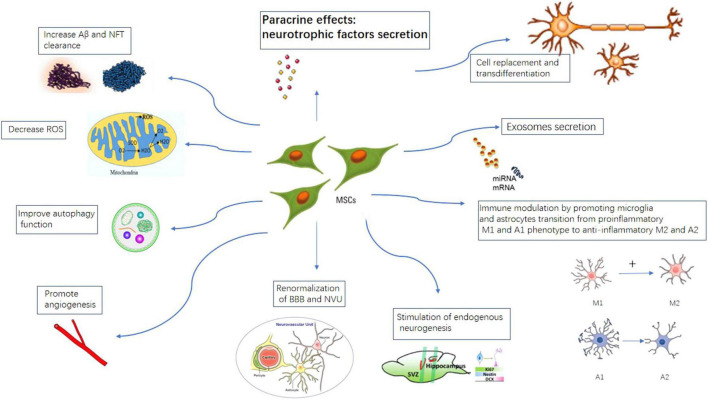
Proposed mechanisms of MSCs therapy in AD. MSCs can take effects by cell replacement, paracrine effects, exosome secretion, immune-modulation, promote angiogenesis, stimulation of endogenous neurogenesis, increase A beta and NFT clearance, improve autophagy, renormalization of blood–brain-barrier.

Different mechanisms for MSCs therapy in AD are illustrated in Figure 4. At present, there are 17 clinical trial studies of mesenchymal stem cell treatment of Alzheimer’s disease registered on the United States clinicaltrial.gov website. A table of current ongoing (and completed studies as well) MSCs clinical trials for AD is listed in Table 1.

**TABLE 1:** Major MSCs clinical trials for AD.

Trial ID	NCT02833792	NCT04482413	NCT03117738	NCT03172117	NCT02054208	NCT01297218	NCT02600130	NCT04388982
Date	06/2016–06/2023	12/2021–12/2023	2017/5–2019/8	05/2017–12/2021	03/2014–12/2019	02/2011–12/2011	10/2016–09/2021	07/2020–08/2022
Sponsors	Stemedica Cell Technologies, Inc.	Nature Cell Co Ltd	Nature Cell Co Ltd	Medipost Co Ltd.	Medipost Co Ltd.	Medipost Co Ltd.	Longeveron Inc.	Cellular Biomedicine Group Ltd.
Country	US	US	US	Korea	Korea	Korea	US	China
Study design	Multi–center, randomized, single–blind, placebo-controlled, crossover study	Randomized, Double–Blind, Active-Controlled	Randomized, Double-Blind, Placebo-Controlled	Quadruple (Participant, Care Provider, Investigator, Outcomes Assessor)	double blind, Single-Center	Open-Label, Single-Center	A Phase, I Prospective, Randomized, Double-Blinded, Placebo-controlled,	Open-Label, Single-Center, Phase I/II
Estimated enrollmen	40	80	21	45	9	9	33	9
Stage	Phase IIa	Phase 2b	phase 1/2	follow up of phase 1/2	Phase 1/2a	Phase 1	Phase 1	Phase 1
Status	Recruiting	Not yet recruiting	Completed with results	Recruiting	Completed with results	Completed with results	Active, not recruiting	Active, recruiting
C ell type	ischem ia–tolerant allogeneic human	Autologous adipose tissue derived	Autologous adipose tissue derived	human um bilicalcord blood derived	human um bilicalcord blood derived	human um bilicalcord blood derived	Longeveron Allogenic Mesenchymal Stem Cells	Allogenic Adipose Mesenchymal Stem
Cell Dosage	1.5 million cells/kg body weight	2.0 × 10^–^8 cells/20 mL of saline with 30% auto-serum .5 m g of Donepezil and AstroStem Placebo; via intravenously AstroStem and Donepezil Placebo every 4 weeks from Week 0 to Week 16	2.0 × 10^–^8 Astrostem cells	Low dose: 1 × 10^–^7 cells/2m L 3 repeated intraventricular administrations via an 0 mm aya Reservoir at 4 week intervals; High dose:3 × 10^–^7 cells	Low dose: 1 × 10^–^7 cells/2m L 3 repeated intraventricular administrations via an 0 m m aya Reservoir at 4 week intervals; High dose:3 × 10^–^7 cells	dose A: 2.5 × 10^–^5 cells/5 uL per 1 entry site, 3 × 10^–^6 cells in total per brain; dose B:6 × 10^–^ 6 cells in total per brain	Low dose: 20 million; High dose: 100 million Longeveron Mesenchymal Stem Cells (LM SC s)	Low Dosage:5μg M SC s–Exos: Mild dosage, 10 μg M SCs–Exos; High dosage, 20 μg M SC s-Exos; Total volume:1ml Frequency:Twice a week Duration:12 weeks
Delivery route	intravenous	intravenous	intravenous	Intraventricular administrations via an 0 mm aya Reservoir	Intraventricular administrations via an 0 m m aya Reservoir	Intraventricular administrations via an 0 mm aya Reservoir	intravenous	intravenous
Outcome measures	primary outcome: SAE; secondary outcome: Changes is scores relatively to baseline using NHSS system	Primary outcome: ADAS-cog; secondary outcome M M SE, A D C S–CG IC, N P I, Treatment related Adverse Events	primary outcom e: SAE; ADAS-cog; secondary outcome:M M SE, A D C S-AD L. C -SSRS, N PIC D R-SO B, G D S	Primary outcome: Number of participants with Adverse event; secondary outcome: Changes from the baseline in ADAS- cog, S-IA D L, K-M M SE, CG A –N PI, ADAS-C og, amyloid beta and tau in cerebrospinal fluid, PIB-PET and FDG -PET at 24 weeks post-dose.	outcome:Number of participants with Adverse event; secondary outcome: Changes from the baseline in ADAS-cog, S-IADL, K-M M SE, CGA-NPI, ADAS-Cog, serum transthyretin, amyloid beta and tau in cerebrospinal fluid, PIB-PET and FDG-PET at 12 weeks post-dose.	outcome: Number of participants with Adverse event; secondary outcome: Changes from the baseline in ADAS-cog, S-IADL, K-M M SE, CGA-NPI, ADAS-Cog, serum transthyretin, amyloid beta and tau in cerebrospinal fluid, PIB-PET and FDG-PET at 12 weeks post-dose.	primary outcome: SAE; secondary outcome: ADAS-Cog 11, M M SE, C SF and Blood inflammatory and A D biomarkers: IL-1,IL-6, TGF-β1,TN F-α,CRP, D-D im er, Fibrinogen, A poE; Brain volume try etc.	30 days FU. No. of adverse events; No. of adverse events 2, 4, 13, 39, and 52 weeks; FU Change from baseline: ADAS-cog, M M SE, adverse events; No. of adverse events 2, 4, 13, 39, and 52 weeks; FU Change from baseline: ADAS-cog, M M SE

*SAE: severe adverse events; ADAS-Cog 11 (Alzheimer’s Disease Assessment Scale-cognitive subscale 11); MMSE (Minimal Mental scale examination); K-MMSE (Korean Minimal Mental scale examination); ADCS-ADL (Alzheimer’s Disease Cooperative Study Activities of Daily Living); ADCS-CGIC (Alzheimer’s Disease Cooperative Study-Clinical Global Impression of Change); QOL-AD (Quality of Life-Alzheimer’s Disease); CDR-SOB (Clinical Dementia Rating-Sum of Boxes); GDS (Geriatric Depression Scale); C-SSRS (Columbia Suicide Severity Rating Scale); NPI (Neuropsychiatric Inventory); CSF(cerebral spinal fluid).*

### Limitations of Current Mesenchymal Stem Cells Trials for Alzheimer’s Disease

Despite the safety being demonstrated in MSCs clinical trials, efficacy has not been proven. We must keep in mind that by the time AD is clinically diagnosed, the neuronal loss and pathological proteins have already accumulated in many brain regions and therefore it is difficult to reverse the disease process. Furthermore, in many clinical trial protocols, patients may receive only several times of stem cells infusion, whereas they indeed might need multiple stem cells infusions over extended period of time. In some trials, autologous MSCs (for example, in those adipose tissue derived MSCs trials) were used. AD patients are usually advanced in age and autologous MSCs may suffer from senescence which compromises their regeneration capability. Most AD clinical trials used intravenous route. The vast majority of intravenously administered stem cells will get detained in the lung and the spleen (Park S. E. et al., 2018) and only very limited number of stem cells can get into the brain. The hostile micro-environment also hindered the survival of infused stem cells. As a result, the paracrine effects of exogenous stem cells can’t compensate for the vast loss of majority neuronal cells in the patient. Anyway, there are many limitations for current MSCs clinical trials which await immediate renovation in the field.

## Conclusion and Future Perspectives

We must look at the pathogenesis of AD from a dynamic point of view. AD has an extremely long latent period before clinical manifestation. It is this long prodromal phase which is most amenable to therapy. Early intervention has the potential to break the vicious cycle of neuronal loss and reverse the clinical course of AD. Therefore, we should make greater efforts to find novel and innovative biomarkers for this asymptomatic phase in AD. For example, disease specific exosomes, microRNAs, blood or CSF early disease biomarkers (especially early biomarkers for neurodegeneration), combined with improved amyloid beta and tau imaging technologies which offer better predictive values for AD (Geekiyanage et al., 2012; Olsson et al., 2016; Hansson et al., 2018; Nakamura et al., 2018).

There is so far no other therapeutic intervention that can have the pleiotropic effects of stem cells. But how to maximize the effect of stem cells to replace the missing nerve cells? In the near future, the development of stem cell based new technologies and related products will drastically change this field. For example, genetic engineering of stem cells can endorse them with stronger neurotrophic effects and greater immune-modulatory effects (Duncan and Valenzuela, 2017). The development of biological scaffolds and other new materials (Kim et al., 2020), nano-sized microvesicles and their modification technologies (Guo et al., 2020) will enable more targeted delivery and more prolonged survival of stem cells in the central nervous system. At the same time, more carefully designed AD clinical trials which target more patients in early phase or prodromal phase of the disease will likely lead to improved results in the near future.

## Author Contributions

JH searched the literature and wrote the review. XW reviewed the manuscript and gave helpful suggestions. Both authors contributed to the article and approved the submitted version.

## Conflict of Interest

The authors declare that the research was conducted in the absence of any commercial or financial relationships that could be construed as a potential conflict of interest.

## Publisher’s Note

All claims expressed in this article are solely those of the authors and do not necessarily represent those of their affiliated organizations, or those of the publisher, the editors and the reviewers. Any product that may be evaluated in this article, or claim that may be made by its manufacturer, is not guaranteed or endorsed by the publisher.
